# A computational investigation on the total cavopulmonary connection circulation assisted by an axial flow pump

**DOI:** 10.3389/fbioe.2025.1626645

**Published:** 2025-08-15

**Authors:** Can Jin, Xudong Liu, Yong Wu, Shengzhang Wang, Junming Zhu

**Affiliations:** ^1^ Anzhen Hospital, Capital Medical University, Beijing, China; ^2^ Department of Aeronautics and Astronautics, Institute of Biomechanics, Fudan University, Shanghai, China; ^3^ Yiwu Research Institute of Fudan University, Yiwu, Zhejiang, China

**Keywords:** Fontan surgery, total cavopulmonary connection, axial flow blood pump, computational fluid dynamics, patient-specific

## Abstract

**Objective:**

Fontan surgery constructs Total Cavo-Pulmonary Connection Circulation (TCPC), but lacks power. Cavopulmonary circulation assist devices (CPAD) has been proposed to support the Fontan circulation. The virtual implantation of blood pumps into the real TCPC structure to analyze the output characteristics of blood pump and flow pattern can better guide design of the pump and the formulation of powered Fontan surgical protocols.

**Method:**

We used computational fluid dynamics (CFD) to explore the power assistance effect of an axial flow blood pump when it was virtually implanted in the real TCPC structures of patients with Fontan circulation and analyzed the differences between constant-speed pumps and variable-speed pumps.

**Results:**

Numerical simulation results showed that the implantation of the blood pump increases pulmonary circulation blood flow by 32.6%, significantly improving the uneven distribution of flow in the left and right pulmonary arteries (the left/right pulmonary artery flow ratio improved from 1:1.8 to 1:1.2). The constant-speed pump mode increased hepatic venous return flow by 19.4% compared to baseline conditions, whereas the variable-speed pump improved it only by 12.7%. The constant-speed pump generated more stable spiral flow patterns in the TCPC structure, reducing energy loss by 21.3% compared to the variable-speed pump, and the wall shear stress distribution in the inferior vena cava (IVC) region was found to be closer to physiological conditions.

**Conclusions:**

According to the results, we confirm that the constant speed mode in powered Fontan circulation has significant advantages in maintaining blood flow stability, optimizing energy efficiency, and promoting organ-specific venous return.

## 1 Background

Fontan palliative surgery has resulted in the effective treatment of a large number of children with functional single ventricles and has prolonged their survival time. However, the constructed cavopulmonary circulation lacks power, and a single ventricle must maintain the power of both the body and lung circulations. Extreme afterload can lead to ventricular hypertrophy and further decrease the pulsatile capacity in a single ventricle, eventually leading to heart failure. To this end, de Leval proposed the concept of a powered Fontan in 1988 (Marc et al., 1988). Over the past 20 years, with the rapid development of ventricular assist devices, research on cavopulmonary circulation assist devices (CPADs) has made great progress. Throckmorton et al. conducted a series of studies on the use of blood pumps to support Fontan circulation (Throckmorton et al., 2007; Throckmorton et al., 2008; Throckmorton et al., 2010), and they presented pressure potential–flow potential output characteristic curves for blood pumps, which provided a basis for the optimal design of pumps (Throckmorton et al., 2011; Throckmorton et al., 2013). Several groups proposed that implantation of blood pumps is a possible direction to achieve powered Fontan circulation (Pradegan et al., 2024). Yang et al. proposed a viscous impeller pump for Fontan circulation support and compared hemodynamic differences in two different blade heights in the pump using CFD simulations and *in vitro* experiments (Yang et al., 2023). Wu et al. proposed a right heart assist device equipped with flexible blades to support Fontan circulation and investigated the optimal elastic modulus of the flexible blades (Wu et al., 2025). Referring to the Jarvik 2000 ventricular assist system (Jarvik, 2014) and the blood pump design scheme of Throckmorton et al., our group optimized the design of a CPAD prototype and its central-core axial blood pump that conformed to the Fontan circulation; virtually implanted it into an idealized TCPC structure; evaluated and analyzed the flow field, pressure, flow characteristics, energy efficiency, blood cell destruction risk, *etc.*, through computational fluid dynamics simulation; and verified the feasibility of the scheme (Liu et al., 2018). However, these studies used an idealized TCPC structure and not a patient-specific structure. In fact, there is a significant difference between the idealized TCPC structure and the real TCPC structure of the patient. The virtual implantation of blood pumps into the real TCPC structure to analyze the output characteristics of the blood pump and flow patterns can better guide the design of the pump and the formulation of powered Fontan surgical protocols. For example, hepatic venous blood flow accounts for 38% of the total abdominal blood circulation, and the impact on Fontan circulation cannot be ignored (Evans et al., 2015). Hepatic vein reflux can induce Fontan-associated liver diseases, such as fibrosis and cirrhosis (Tellez et al., 2018; Trusty et al., 2018). Therefore, the improvement of the reflux of the hepatic venous after CPAD implantation can be used to evaluate the clinical value of CPAD implantation.

In this study, the TCPC geometry model of the real Fontan circulation was selected for the virtual implantation of CPAD, and the changes in hemodynamic parameters of patients before and after axial flow blood pump implantation were analyzed through computational fluid dynamics simulation; the output characteristics of the blood pump in this state were evaluated, and the feasibility of CPAD implantation to assist Fontan circulation was clarified. In addition, the variable-speed blood pump was studied; the right heart to the pulmonary artery ejection process was simulated by the change in the rotation speed, and the difference in hemodynamic indexes between the constant-speed pump and the variable-speed pump in the real Fontan circulation was compared and analyzed to clarify the feasibility and physiological value of the variable-speed design of the axial flow blood pump.

## 2 Materials and methods

### 2.1 Geometrical model

**FIGURE 1:**
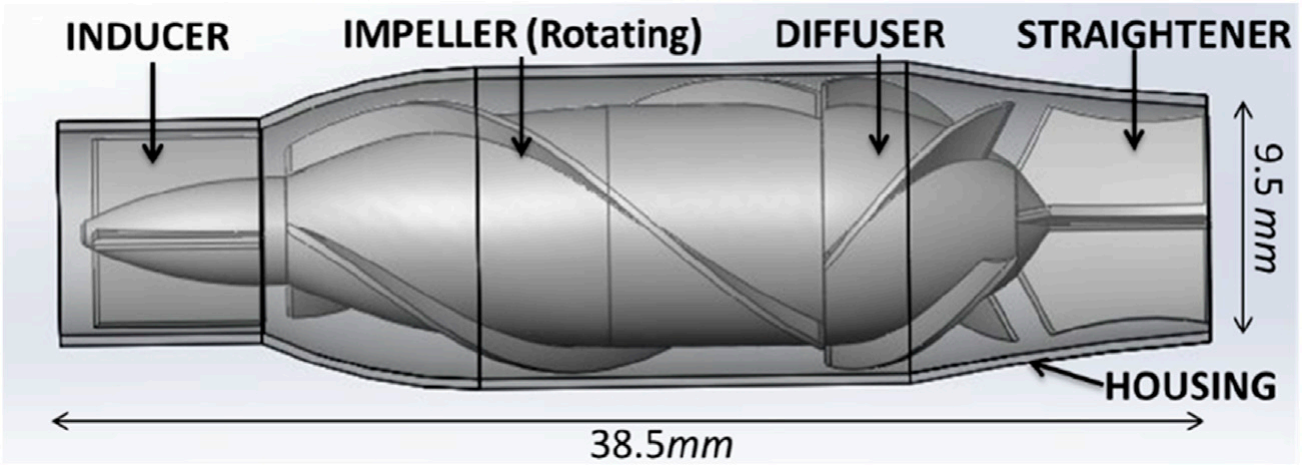
Diagram of the blood pump structure. The structure of the axial flow pump includes a housing, inducer, impeller, diffuser, and straightener.

The Fontan circulation assist device in this study used an axial flow blood pump previously designed by our group (Liu et al., 2018). The blood pump was optimized with reference to the Jarvik 2000 design; the size and output characteristics of the pump were adjusted, and the systemic circulation output parameters of the blood pump were adjusted to better align with the requirements of cavopulmonary circulation assist, as shown in Figure 1. The TCPC model was derived from a 13-year-old female patient with a single-ventricular cardiovascular malformation, who underwent a Fontan surgery with a 12-mm artificial vessel matching the diameter of the pulmonary artery. The raw DICOM data of the patient’s heart were collected using a Siemens CT, and the inferior vena cava (IVC) and hepatic vein were scanned at the same time.

**FIGURE 2:**
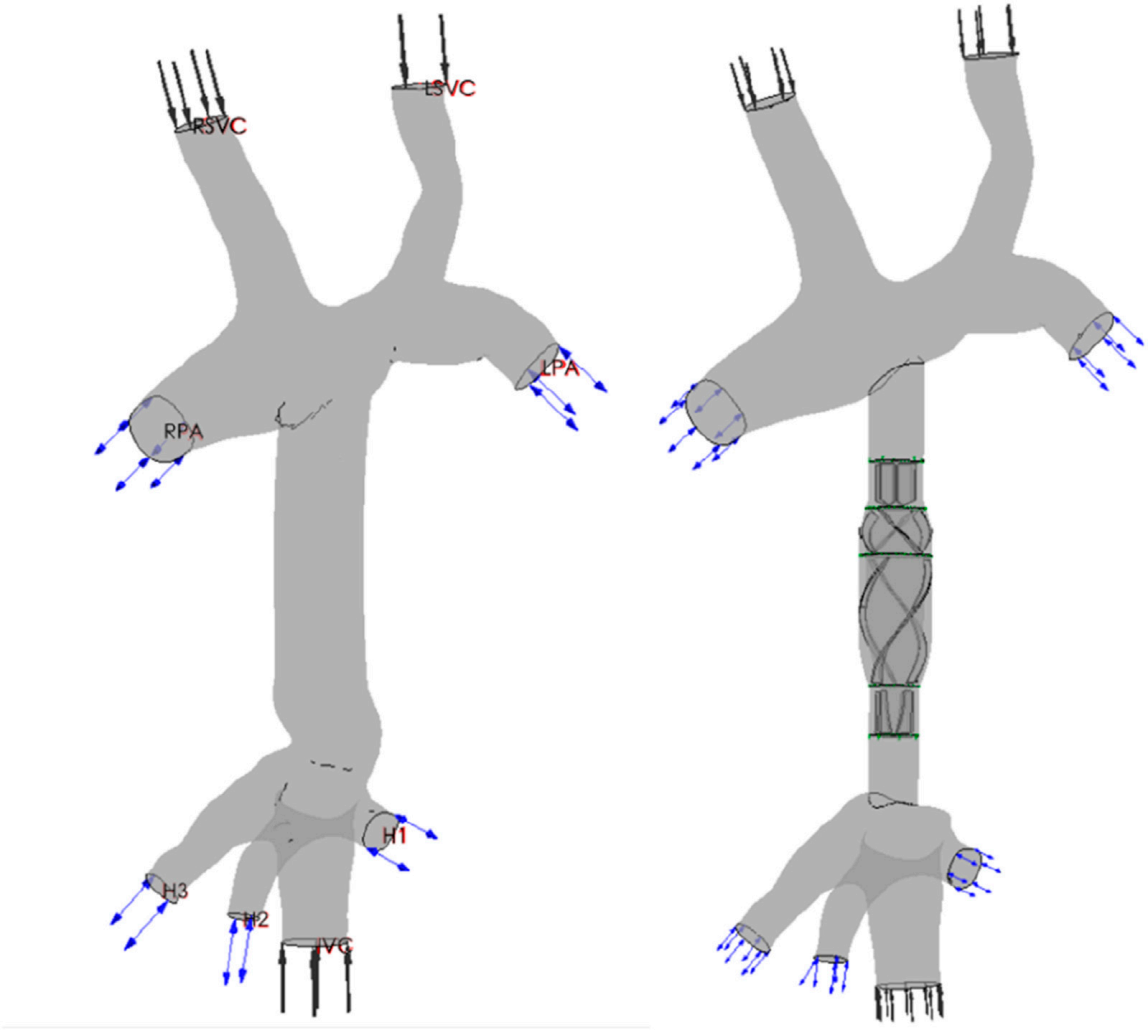
Renderings of the TCPC structure and CPAD virtual implantation in a Fontan patient: the anatomical structure of the TCPC (left figure) and the implantation effect of the CPAD in the TCPC (right figure). The TCPC structure includes the inferior vena cava (IVC), the superior vena cava (RSVC and LSVC), the two pulmonary arteries (RPA and LPA), and the three hepatic veins (H1, H2, and H3).

DICOM data obtained from the CT scans were reconstructed using Mimics 17.0 (Materialise, Leuven, Belgium) through region segmentation and region growth to obtain a preliminary 3D TCPC model. The model centerline was extracted, and the model was smoothed using Geomagic Studio 13.0 (Geomagic, Dallas, United States). The NURBS surfaces of the smoothed model were generated and exported. The CPAD was virtually implanted according to the centerline of the artificial vascular model, and the specific process was as follows: (1) the TCPC geometry model was imported into Geomagic studio 13.0, and the reasonable position of the axial blood pump on the centerline of the outer pipeline was determined according to the direction of the centerline; (2) the artificial vascular model in the blood pump implantation region was deleted to obtain the superior vena-pulmonary artery region and the inferior vena cava–hepatic vein region; (3) because the diameter of the artificial vascular model in the inferior vena cava did not match the diameter of the inlet and outlet of the axial blood pump, it was necessary to modify the artificial vascular model. The artificial vascular interface partially remained, and the models were imported into SolidWorks 2015 (Dassault Systemes, Massachusetts, United States) for vascular growth; (4) a scanning trend line is established between the artificial vascular model and the blood pump entrance and exit through the section normal and the centerline, and the artificial vascular model connecting with the axial blood pump is completed through surface scanning under the centerline guide; and (5) the superior vena cava and inferior vena cava were assembled with the axial blood pump, and the construction of the pump–TCPC was completed. The effect of the virtual implantation of CPAD in a real Fontan circulation, before and after implantation, is shown in Figure 2.

### 2.2 Mesh generation

The computational grid of the pump–TCPC model was generated using ICEM-CFD 15.0 (Ansys, Canonsburg, United States). The main body of the fluid domain was meshed using tetrahedral elements. The boundary layer was meshed using prism elements with five layers to better describe the flow field near the wall, so that the calculation of the hemodynamic parameters of the wall was more reasonable. Taking the superior vena cava model of this case as an example, a grid independence test was carried out. The evaluation criterion is the energy loss after the fluid flows through the region (Bove et al., 2007), which is expressed in Equation 1:

$$\begin{array}{l} {\dot{E}}_{\text{loss}}=E_{\text{inlet}}-E_{\text{outlet}}\\ =\sum_{\text{inlet}}\left(P_{i}+\frac{1}{2}\rho V_{i}^{2}\right)Q_{i}-\sum_{\text{outlet}}\left(P_{o}+\frac{1}{2}\rho V_{o}^{2}\right)Q_{o}. \end{array}$$ (1)

The results of the obtained grid independence test are shown in Table 1. With the increase in the number of grid nodes and elements, the difference in energy consumption indicators gradually decreased, and the energy consumption indicators tended to converge. At a maximum grid size of 0.6, the deviation of the energy consumption index decreased to less than 2%. As the maximum grid size decreased further, it converged to 0.47%. The independence of the grid was verified. Therefore, the grid with a maximum size of 0.6 was selected. The number of nodes was 181,930, and the number of grid elements was 672,734. Other vascular grids also referred to this standard, and the maximum grid size of 0.6 was selected as the basis for grid generation. The axial blood pump was meshed using hexahedral elements, with a maximum grid size of 0.7. The corresponding number of nodes was 361,581, and the number of elements was 1,332,324.

**TABLE 1:** Grid independence test for the superior vena cava model.

Maximum element size	No. of nodes	No. of elements	Energy gain (mW)	(E−Eo)/Eo·100%
1.4	7,808	18,849	4.913937	
1.2	14,855	37,389	4.5491145	7.42
1	20,874	54,419	3.499976	23.06
0.8	47,991	102,173	3.825639	9.30
0.7	106,455	397,902	3.745639	2.09
0.6*	181,930*	672,734*	3.678674	1.79
0.5	321,494	1,049,365	3.69588	0.47

### 2.3 Numerical scheme

**FIGURE 3:**
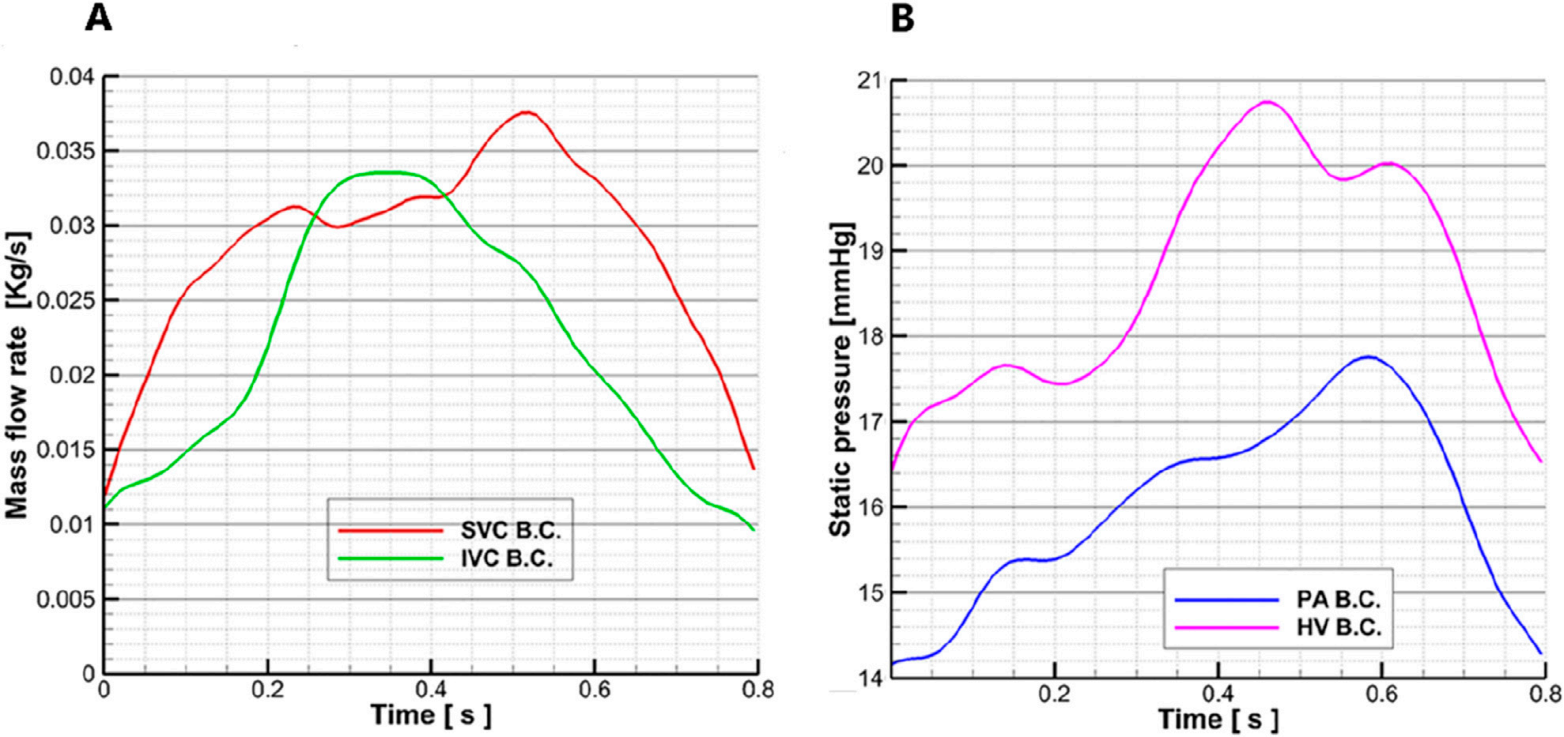
Flow–time curves and pressure–time curves are measured: **(A)** the measured flow–time curve of the superior vena cava and inferior vena cava in a Fontan patient; the red line represents the superior vena cava flow waveform, and the green line represents the inferior vena cava flow waveform. **(B)** Measured pressure–time curve of the pulmonary artery and hepatic vein in a Fontan patient; the pink line represents the hepatic vein pressure waveform, and the blue line represents the pulmonary artery pressure waveform.

The flow and pressure waveforms used in this study were clinically measured after Fontan surgery was performed on a 13-year-old female patient with a single ventricle, and the specific methods are as follows: the flow velocity–time curves of the superior vena cava and the inferior vena cava were obtained through color ultrasound, and the flow rate curve envelope matching the phase of the ECG signal was automatically drawn by ECG signal gating to realize the extraction of the two curves. The flow–time curves were obtained by combining the flow velocity–time curves with the diameter of the superior and inferior vena cava and blood density, respectively. The pressure–time curves at the pulmonary artery and inferior hepatic vena cava were measured through cardiac catheterization. According to the ECG monitoring signal, the pressure–time curve matched the phase of the flow velocity–time curve. The measured flow–time and pressure–time curves for the real Fontan circulation are shown in Figure 3.

The blood vessel wall is set as a rigid wall, and the wall surface has no slip and no penetration. The density of the blood is 1,050 kg/m3, the dynamic viscosity is 0.0035 Pa∙s, and the specific heat capacity is 3.594 kJ/kg/K. ANSYS CFX 15.0 (Ansys, Canonsburg, United States) was used to solve the Navier–Stokes equation for the transient solution, and the RANS model and the standard k-epsilon model were applied. The average Reynolds number was 8,400. The inlet of the superior vena cava was taken as the flow boundary condition, and the flow waveform is shown by the red line in Figure 3A. In this case, a superior vena cava shunt was used, in which the flow distribution ratio of the superior vena cava inlet was LSVC:RSVC = 40%:60% (Downs et al., 2012). The inferior vena cava inlet was also set as a flow boundary condition, and the waveform is shown by the green line in Figure 3A. To clearly present the regurgitation in the inferior hepatic vena cava, the hydrostatic opening boundary conditions were set at the three hepatic veins (LHV, MHV, and RHV) in the inferior lumen, and the pressure waveform was represented by the pink line in Figure 3B. The open boundary condition was set to clearly capture the differences in the distribution of blood flow in the hepatic vein between the model of the Fontan circulation and the pump–TCPC model. To analyze the effect of blood pump implantation on the distribution of blood flow to the left and right lungs, an open hydrostatic boundary condition was set at the LPA and RPA outlets, and the waveform is shown by the blue line in Figure 3B.

**FIGURE 4:**
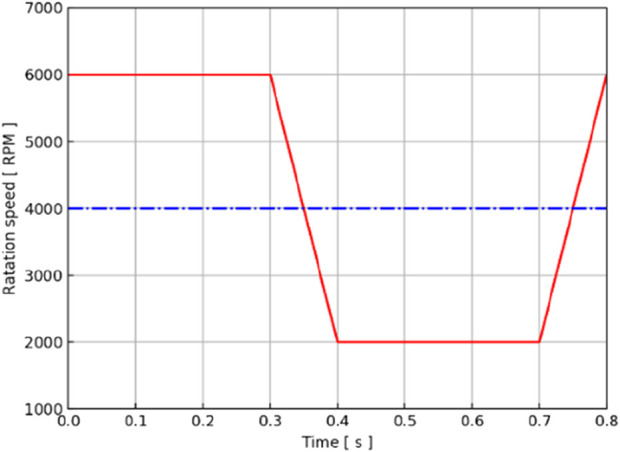
Rotation speed–time curve of the axial blood pump in a cardiac cycle. The red line represents the waveform of the variable-speed pump, and the blue line represents the baseline of the constant-speed pump.

Figure 4 shows how the constant and variable speeds of the axial blood pump were set for a cardiac cycle. Through previous studies, it was found that the pressure–flow output characteristics and energy indicators of the axial blood pump at 4,000 RPM could meet the output requirements of the Fontan circulation (Liu et al., 2018), so 4,000 RPM was used as the speed setting of the constant speed pump. For the variable-speed pump, the previous research conclusion showed that the speed of 2,000 RPM∼6,000 RPM was the more suitable output range for the axial flow pump (Liu et al., 2018), so the trapezoidal wave was used as the waveform of the variable-speed pump. Taking 0.8 s as an example, 0 s–0.3 s was taken as the 6000 RPM high-speed rotation segment to simulate right cardiac isobaric contraction ejection. In addition, 0.3 s–0.4 s was a fast deceleration section, which changed from high speed to low speed, simulating the isochoric relaxation of the right heart; 0.4s–0.7 s, 2000 RPM low-speed rotation section, was comparable to isobaric relaxation of the right heart; 0.7 s–0.8 s, for the rapid acceleration stage, from low speed to high speed, was equivalent to the iso-volume contraction of the right heart, to prepare for the pumping of the next cardiac cycle. This waveform setting ensured the same average rotational speed as the constant-speed pump in one cardiac cycle, aligning the mechanical power provided by the two cases for the Fontan circulation. Trapezoidal waves were used to approximate right-sided equivalent output, providing pulsatile blood flow to the Fontan circulation that was relatively close to physiological conditions.

According to the patient’s pulse of approximately 75 BPM, a cardiac cycle of 0.8 s was determined. A transient simulation was carried out in two cardiac cycles of 1.6 s and a step size of 0.005 s, and the simulation results of the second cardiac cycle were selected for the analysis. The criterion for calculating convergence is RMS<10^–4^. The original TCPC model, the constant-speed pump–TCPC model, and the variable-speed pump–TCPC model were simulated. The simulation results of three models (original model, constant-speed model, and variable-speed model) were analyzed, and the pressure–flow output characteristics, energy index, flow patterns, and flow distribution were compared.

## 3 Results

### 3.1 Output characteristics of the blood pump in the TCPC cycle

**FIGURE 5:**
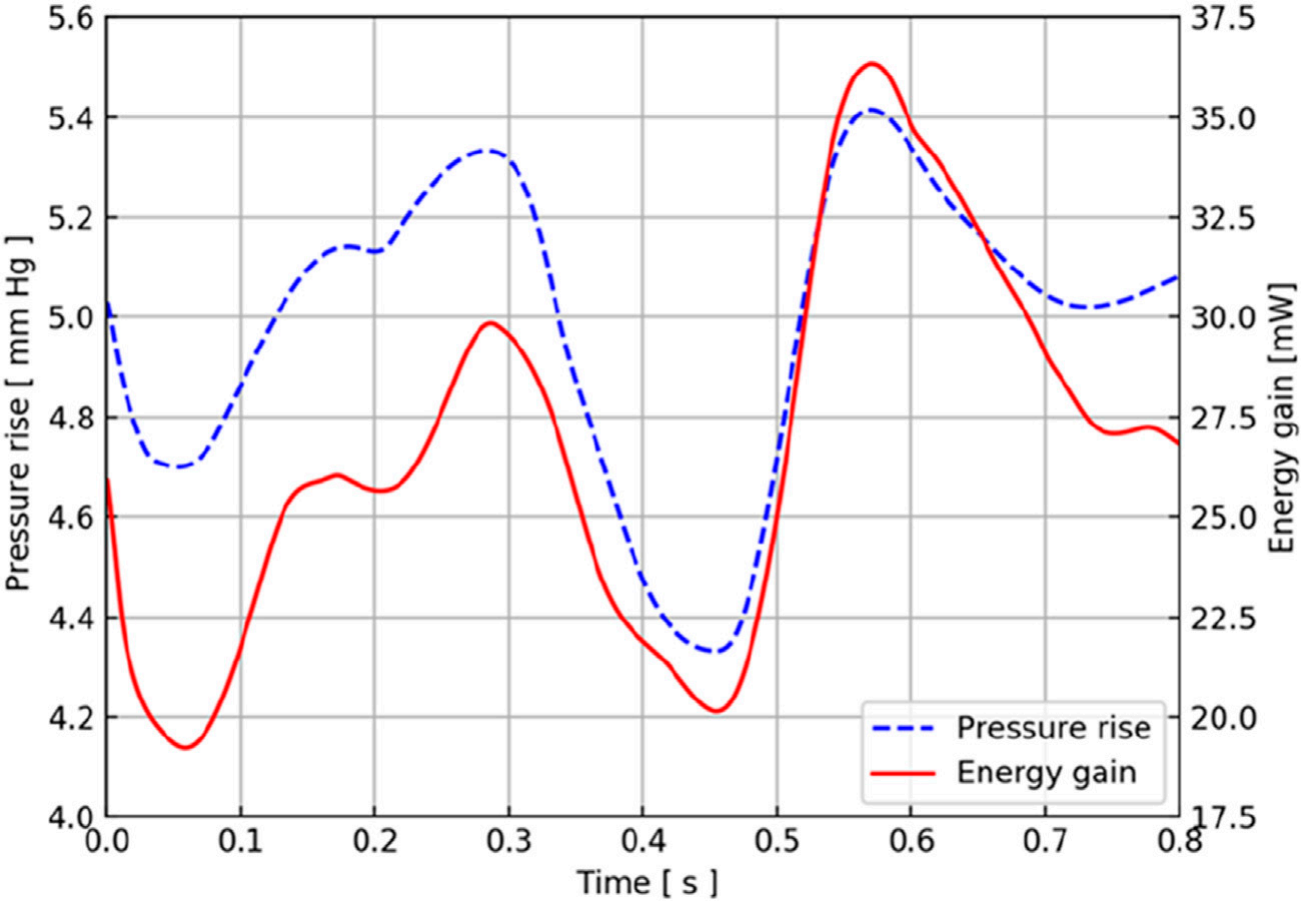
Pressure and energy output characteristic curves of the CPAD implanted in the real TCPC structure. The blue dotted line represents the pressure waveform, and the red solid line represents the energy waveform.

According to the CFD simulation results, the pressure increase after the axial flow blood pump was calculated, and the relationship between the pressure output of the blood pump and time in the TCPC structure of this patient was obtained, as shown by the blue dotted line in Figure 5. The output of the constant-speed pump was found to be relatively stable, and the pressure increase provided by the axial blood pump was observed to be between 4 mmHg and 6 mmHg in one cardiac cycle. Two maximums were obtained at approximately 0.3 s and 0.55 s, and two minimums were obtained at approximately 0.05 s and 0.45 s.

Considering that the axial flow blood pump brings an increase in energy to the entire TCPC structure, the energy output of the blood pump in this TCPC structure is shown by the red solid line in Figure 5. During a cardiac cycle, the energy provided by the axial blood pump for the entire TCPC structure was approximately 20 mW–40 mW. The two maximums appeared at 0.3 s and 0.55 s, and the minimums appeared at approximately 0.05 s and 0.45 s. The time at which these extreme points were obtained coincided with the pressure curve.

Because the implantation location of the blood pump is the inferior vena cava, the flow of the inferior vena cava has a direct impact on the output of the blood pump pressure and energy. The blood flow waveform at the inferior vena cava inlet showed that it reached the maximum value at 0.3 s, and the blood flow of the superior vena cava reached the maximum value at 0.55 s, which is consistent with the acquisition of the two extreme points. The blood flow in the superior vena cava at approximately 0.45 s also obtained a maximum similar to that at 0.55 s.

### 3.2 Effect of blood pump output characteristics on flow patterns and flow distribution

**FIGURE 6:**
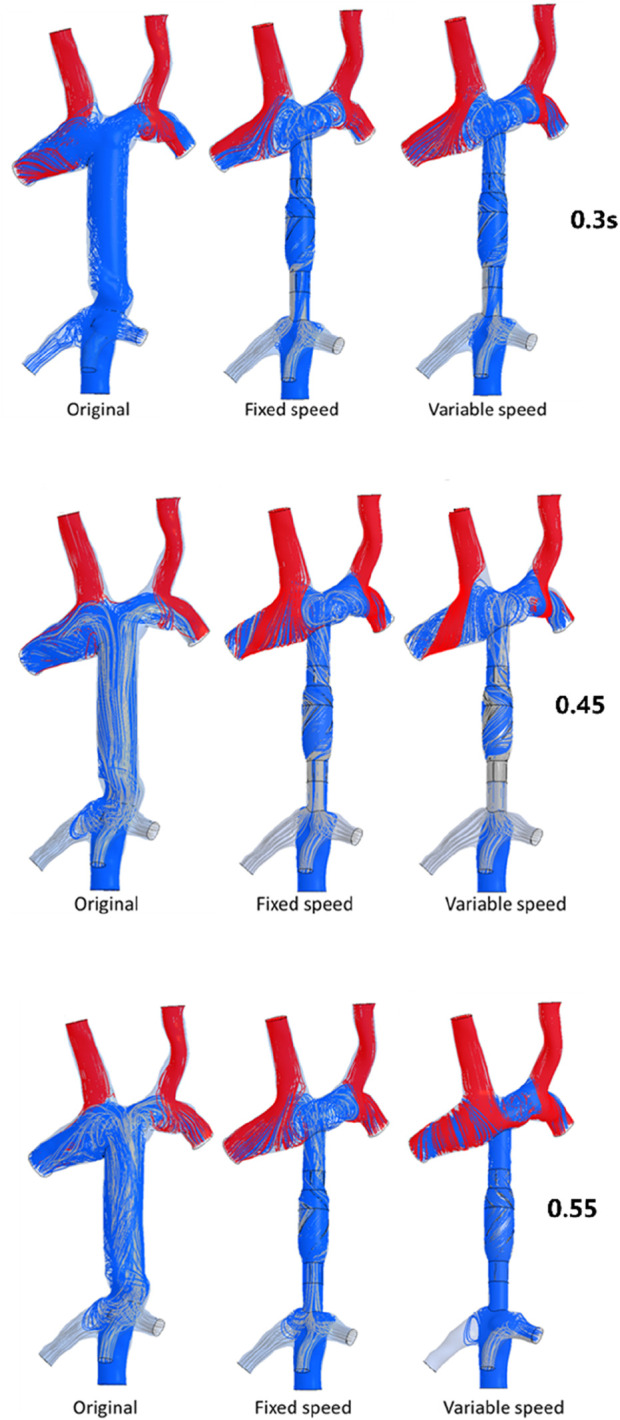
Flow patterns and flow distribution of three models at 0.3 s, 0.45 s, and 0.55 s. The first column presents the original model of the TCPC structure; the second column presents the model of TCPC implanted with the constant-speed pump; the third column presents the model of TCPC implanted with a variable-speed pump; and the blue streamline represents the flow from the IVC, the red streamline represents the flow from the SVC, and the gray streamline represents the flow from the hepatic vein.

To analyze the influence of the implantation and output characteristics of the axial flow pump on the hemodynamic parameters of the Fontan circulation, flow patterns and flow distribution were analyzed at 0.3 s, 0.45 s, and 0.55 s, when the pressure and energy indexes were taken to the extreme points. The differences in flow patterns and flow distribution among the original model without a blood pump, the constant-speed model, and the variable-speed model with a blood pump were compared to obtain a more reasonable design reference for blood pump output characteristics Figure 6 shows the flow patterns and flow distribution among the original, fixed, and variable models at 0.3 s, 0.45 s, and 0.55 s.

**FIGURE 7:**
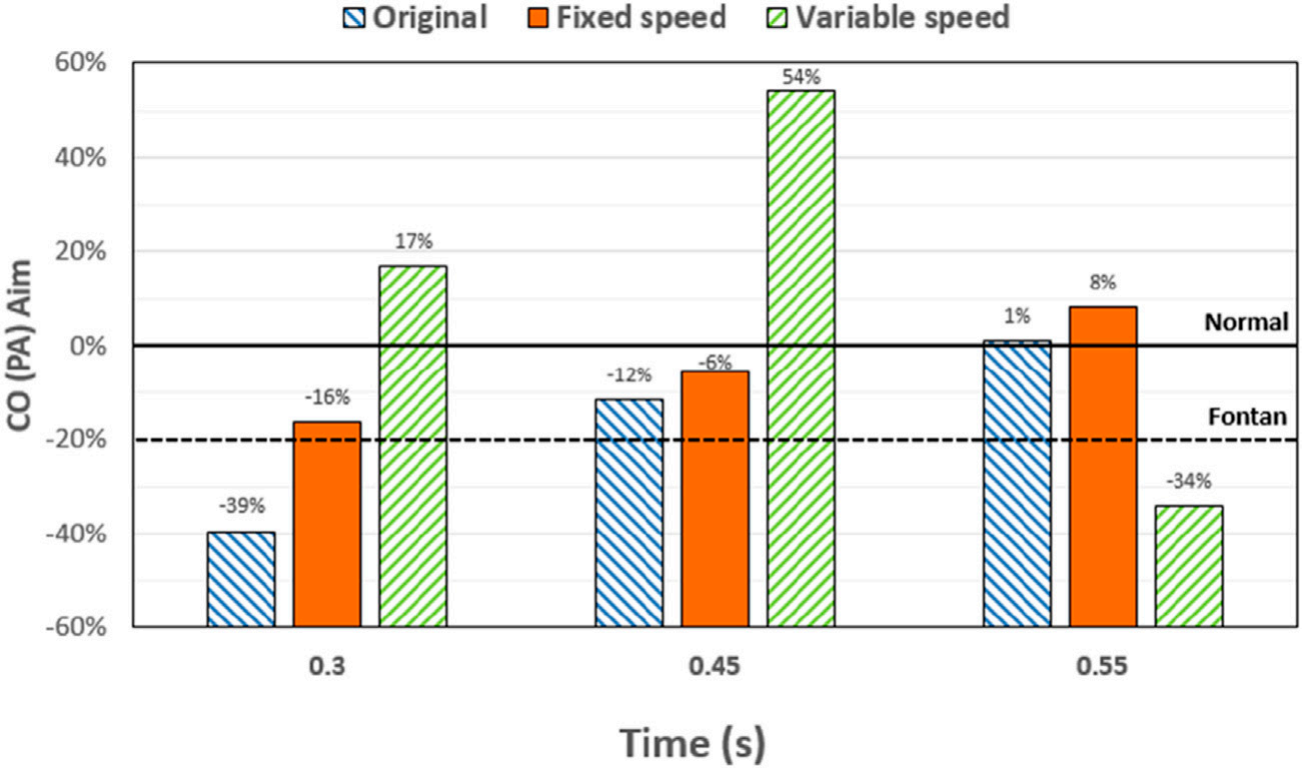
Percentage deviation between the target cardiac output (total blood flow into the lungs) and the baseline value for the three models at different times. The blue right slash represents the original model, the orange represents the constant-speed model, and the green left slash represents the variable-speed model; the black dotted line represents the design target of the Fontan surgery, and the solid black line represents the normal value of the human body.

**FIGURE 8:**
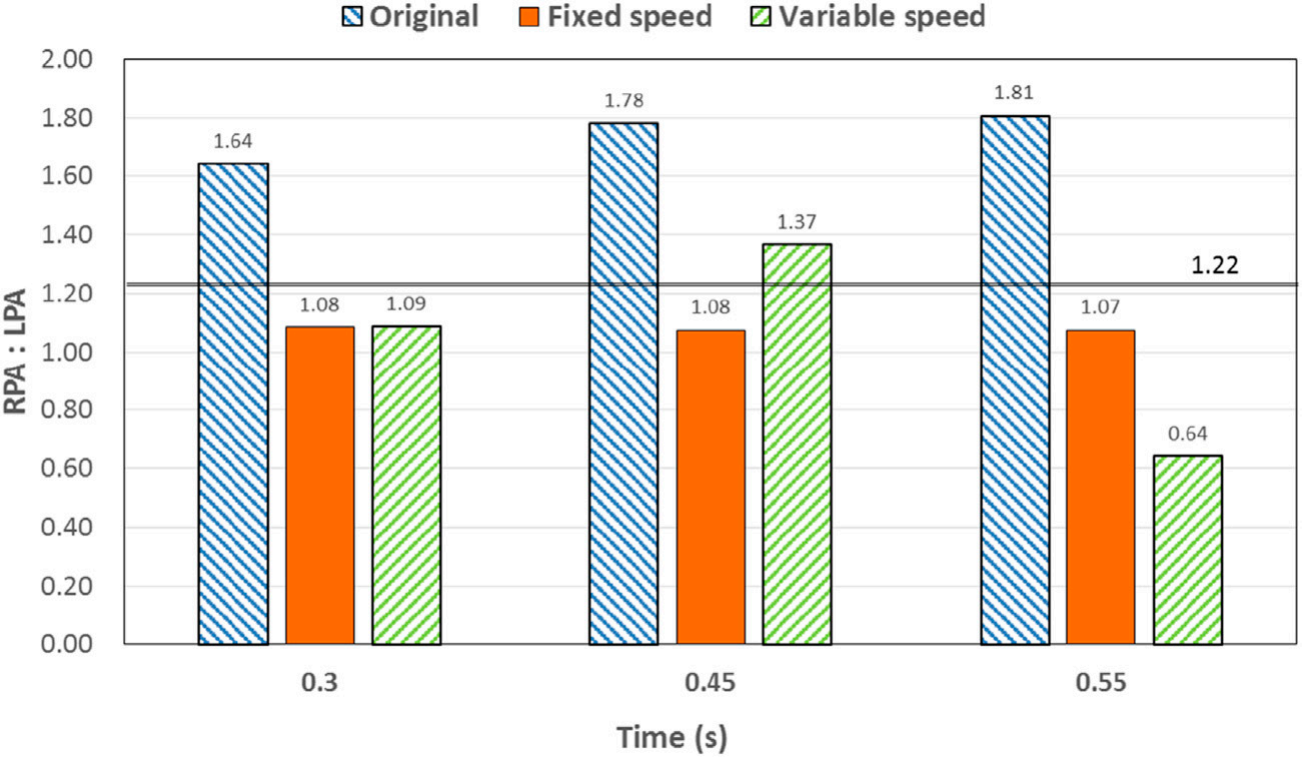
Percentage deviation between the left and right pulmonary artery flow distribution and the normal value of the three models at different times. The blue right slash represents the original model, the orange represents the constant-speed model, and the green left slash represents the variable-speed model. The solid black line represents the normal value of the human body.

**FIGURE 9:**
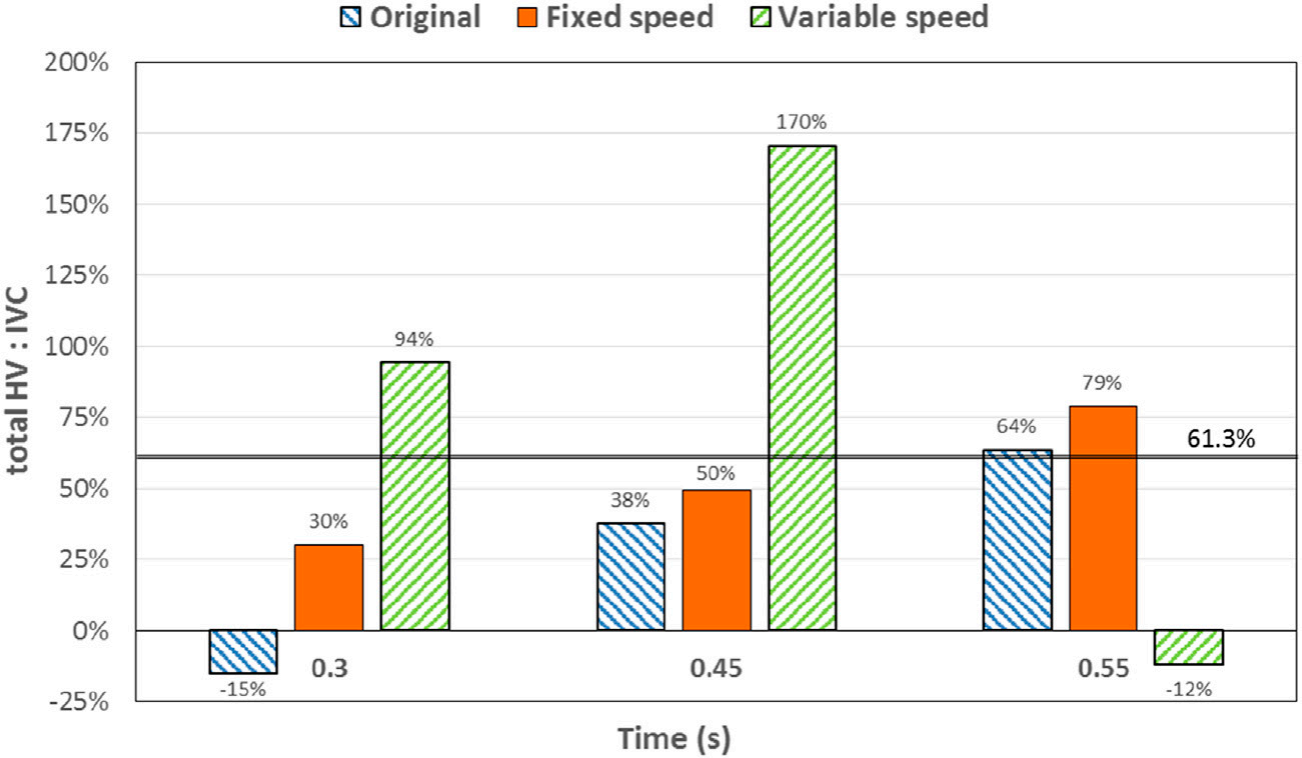
Percentage deviation between the total hepatic venous flow/IVC inlet flow and the normal value of the three models at different times. The blue right slash represents the original model, the orange represents the constant-speed model, and the green left slash represents the variable-speed model. The solid black line represents the normal value of the human body.

**FIGURE 10:**
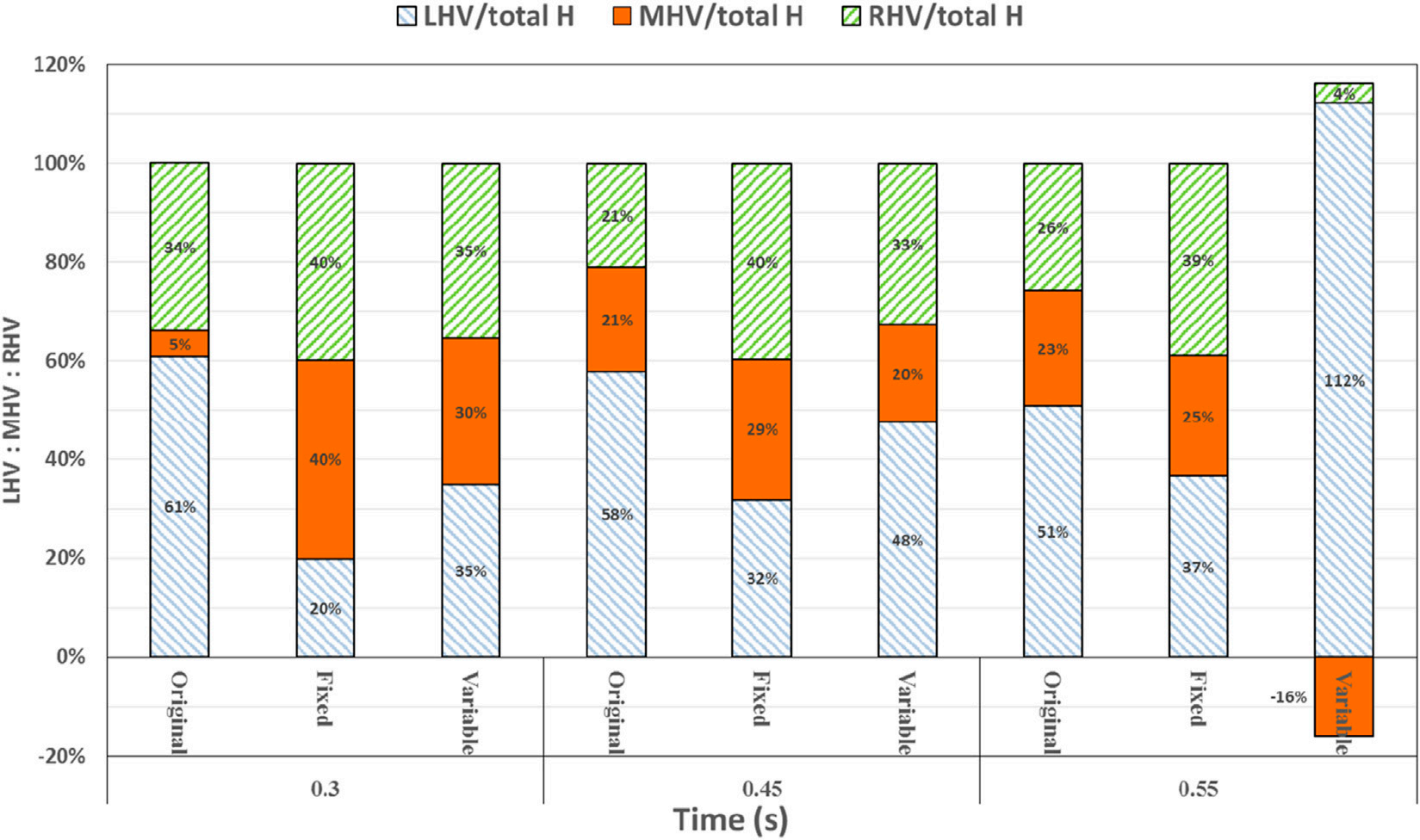
Percentage deviation between the left, middle, and right hepatic venous flow distribution of the three models and the normal value. The blue right diagonal line represents the proportion of left hepatic venous flow, the orange line represents the proportion of middle hepatic venous flow, and the green left oblique line represents the proportion of right hepatic venous flow.

At 0.3 s, when the blood flow rate of the IVC reached the maximum, the flow field in the original, constant, and variable speed models is shown in the first row of Figure 6. A small amount of regurgitation was evident at all three hepatic veins in the IVC in the original model. As the inlet flow of the IVC was at its maximum and the pressure at the hepatic vein was not high, the flow was distributed by the IVC to the LHV, MHV, and RHV. As shown in Figure 9, the ratio of the total hepatic venous flow to the IVC inlet flow was −15%. In other words, the hepatic vein is used as the outlet so that the blood flow in the IVC was −15% of the original IVC flow, and as shown in Figure 7, there is a 39% gap between the return blood volume and the design goal of pumped Fontan circulation. The distribution ratio of blood flow into the lungs to the left and right lungs is shown in Figure 8, and the original flow ratio into the left and right lungs was 1:1.64. The distribution of flow between the three hepatic veins is shown in Figure 10, with the original model having a ratio of 34:5:61 and an uneven distribution, where blood flow in the MHV is concentrated in the RHV.

Through the flow patterns of the constant-speed model at this time, it could be observed that the implantation of the axial flow pump eliminated the hepatic venous regurgitation at the time of maximum IVC flow; the LHV, MHV, and RHV were turned into inlet boundary conditions, and the blood flow of the IVC increased; and the hepatic venous flow showed a certain amplification effect on the IVC flow, amplifying it by 30%, which basically achieved the design goal of pumped Fontan circulation. However, there remained a 16% gap with the total flow rate into the lungs of a normal human body, and the ratio of left and right lung flow was 1:1.08, which is close to the normal human level of 1:1.22 (Chaloupecký et al., 2005). The distribution of flow among the hepatic veins was 40:40:20, which was more uniform than that of the original model.

For the variable-speed model late stage of high-speed working corresponded to the right heart isovolumetric ejection phase, at which point the blood pump output capacity remained at a high level, resulting in a further increase in blood flow from the inferior vena cava to the liver. The total pulmonary blood flow exceeded normal human indicators by approximately 17%, which can lead to insufficient hepatic blood flow in Fontan patients, resulting in postoperative liver function decline. At the same time, the left-to-right pulmonary flow ratio was 1:1.09, and the blood flow distribution among the hepatic veins was 35:30:35, further achieving a reasonable distribution of the hepatic venous flow.

At 0.45 s, when the output characteristic curve of the constant-speed pump reached its minimum, the flow patterns of the original, constant-, and variable-speed models are shown in the second row of Figure 6. Hepatic venous regurgitation was absent in all three models. The hepatic venous blood flow distribution, hepatic venous blood flow proportion, and pulmonary blood flow in the original model and the constant-speed model were significantly improved compared with the previous time, and the constant-speed model was still better than the original model in the lung blood flow distribution. It could be concluded that when the pump is constantly outputting at 4,000 RPM, the low-output condition was better than the high-output condition to obtain a hemodynamic index that matches the TCPC structure. For the variable-speed model, there were anomalous changes in all metrics. At this time, the speed of the pump has just passed through the rapid deceleration phase (corresponding to the right heart isochoric diastolic stage), and the physiological flow patterns were not fully adapted to this change, so there will be abnormal indicators. This also corroborates the rapid change in the pump speed, which may lead to the deterioration of the local hemodynamic environment.

At 0.55 s, the flow from the SVC reached a maximum value, whereas the output characteristics of the constant-speed pump were taken to obtain the maximum value, and the flow patterns in the original, constant-speed, and variable-speed models are shown in the third row of Figure 6. At this time, the LHV of the variable-speed axial flow pump model showed regurgitation, whereas the RHV allocated a very small amount of flow, accounting for only 4% of the flow of the three hepatic veins; the distribution of blood flow to the LHV in the MHV also occurred, and other hemodynamic indicators were further deteriorated. At this point, the variable-speed pump had been operating at a low speed of 2,000 RPM for a long time, and the deformed parameters reflected that the speed was too low and did not match the hemodynamic characteristics of the TCPC structure. The flow patterns of the constant-speed pump also improved compared with the previous moment, and the blood flow into the lungs could reach the normal human level. The flow supplement from hepatic venous to the IVC was also more reasonable, and the hepatic venous blood flow distribution was maintained at the same level.

From the flow patterns, it could be observed that the implantation of the blood pump into the IVC brought an obvious swirling flow field to the TCPC SVC anastomosis, which effectively reduced the possibility of thrombosis and assisted in the repair of damaged endothelial cells.

## 4 Discussion

The total pulmonary flow obtained by substituting the boundary conditions of the Fontan model into the normal human pulmonary circulation was taken as the design goal of the total blood flow (or cardiac output) into the lungs of the Fontan surgery, and the ultimate goal of the implanted CPAD was to completely simulate the right heart output of the normal human body, so this standard was adopted to analyze the advantages and disadvantages of the power-assisted Fontan circulation. At the same time, considering the decrease in the systemic blood flow caused by the cardiac vascular malformation of Fontan patients and the resulting hypoplasia of some organs, the design goal of the power-assisted Fontan circulation plan was 80% of the normal human body with reference to the existing Fontan surgical plan (Baretta et al., 2012; Pekkan et al., 2005). Figure 9 shows the ratio of the total hepatic vein blood flow to IVC inlet flow, and the reference values selected in the figure are based on the fact that the design target of the total hepatic venous blood flow to the IVC flow in the Fontan surgery is 61.3% and that the normal human hepatic vein accounts for approximately 38% of the inferior vena flow before it flows into the inferior vena cava. Figures 9, 10 illustrate pulmonary and hepatic blood flow distribution, respectively. The distribution ratio of left and right pulmonary artery flow in the normal human body is LPA:RPA = 45:55, which is related to the physiological basis that the left lung has two lobes and the right lung has three lobes due to a left-sided cardiac mass; therefore, RPA:LPA = 1.22 is taken as the design criterion. The implantation of the constant-speed axial flow pump eliminated the hepatic venous regurgitation at the time of maximum IVC flow, and the LHV, MHV, and RHV were transformed into inlet boundary conditions; the blood flow of the IVC returned to the heart increased, and the hepatic venous flow showed a certain amplification effect on the IVC flow, which basically achieved the design goal of powered Fontan circulation. Meanwhile, the flow distribution among the hepatic veins is 40:40:20, which is more uniform than that of the original model. The variable-speed pump made the total blood flow into the lungs of the hepatic venous blood flow in the IVC exceeded the normal human body value by approximately 17% under the condition of high speed, which is easy to cause insufficient hepatic blood flow for Fontan patients, resulting in the decrease in postoperative liver function (Daniels et al., 2017, Kaulitz et al., 2014).

This study has several limitations. The external conduit of TCPC in this study is a straight tube, but the external conduit of the Fontan patient may be curved; therefore, the flow pattern and flow distribution may be different from that of a straight tube when the axial hemorrhage pump is implanted in a curved tube. This study did not analyze the risk of hemolysis after implantation of the constant-speed and variable-speed axial flow pumps. The surgical implantation method and power supply method of the axial flow blood pump were discussed. The results and conclusions of this study have not been validated through *in vitro* or animal experiments. In future works, an *in vitro* experimental platform of TCPC will be constructed by 3D printing, and a real axial flow pump will be implanted into the physical model to validate the numerical results of this study.

## 5 Conclusion

In this study, an axial flow blood pump with two kinds of working states, constant-speed mode and variable-speed mode, was virtually implanted into a real TCPC geometry through virtual surgery, and the flow and pressure data measured in real cases were imposed as boundary conditions to simulate the blood flow of the entire pump–TCPC in a cardiac cycle, and the pressure and energy output characteristic curves of the blood pump in this structure, the improvement in the flow patterns, and the flow distribution after blood pump implantation were analyzed and compared. The following main conclusions were obtained: a) the implantation of the axial flow pump into the Fontan circulation is effective; it can increase blood flow into the lungs, improve the distribution of blood flow in the left and right pulmonary arteries, and promote the reflux of the hepatic veins; b) compared with the variable-speed pump, the TCPC structure after the implantation of the constant-speed pump has significant advantages in flow field characteristics and flow distribution; and c) after implantation of the axial flow pump, a significant swirling flow field was introduced at the anastomosis of the SVC of the TCPC, which could reduce the likelihood of thrombosis.

## Data Availability

The raw data supporting the conclusions of this article will be made available by the authors, without undue reservation.
